# Covariation between Spike and LFP Modulations Revealed with Focal and Asynchronous Stimulation of Receptive Field Surround in Monkey Primary Visual Cortex

**DOI:** 10.1371/journal.pone.0144929

**Published:** 2015-12-15

**Authors:** Kayeon Kim, Taekjun Kim, Taehwan Yoon, Choongkil Lee

**Affiliations:** 1 Department of Psychology, Seoul National University, Gwanak, Seoul, Korea; 2 Program of Cognitive Science, Seoul National University, Gwanak, Seoul, Korea; CSIC-Univ Miguel Hernandez, SPAIN

## Abstract

A focal visual stimulus outside the classical receptive field (RF) of a V1 neuron does not evoke a spike response by itself, and yet evokes robust changes in the local field potential (LFP). This subthreshold LFP provides a unique opportunity to investigate how changes induced by surround stimulation leads to modulation of spike activity. In the current study, two identical Gabor stimuli were sequentially presented with a variable stimulus onset asynchrony (SOA) ranging from 0 to 100 ms: the first (S1) outside the RF and the second (S2) over the RF of primary visual cortex neurons, while trained monkeys performed a fixation task. This focal and asynchronous stimulation of the RF surround enabled us to analyze the modulation of S2-evoked spike activity and covariation between spike and LFP modulation across SOA. In this condition, the modulation of S2-evoked spike response was dominantly facilitative and was correlated with the change in LFP amplitude, which was pronounced for the cells recorded in the upper cortical layers. The time course of covariation between the SOA-dependent spike modulation and LFP amplitude suggested that the subthreshold LFP evoked by the S1 can predict the magnitude of upcoming spike modulation.

## Introduction

Visual events are broken down into local signals by retinal cells, and subsequent integration of these signals across visual space constitutes a critical element of central visual processing. Accordingly, the receptive fields (RFs) of cells in the later stages of processing are increasingly large, suitable for integrating signals from wider regions of the visual space. However, numerous studies on surround interactions have shown that even at the very early stages of processing, integration of visual signals across a wide region of visual space can occur for neurons with small RFs. For example, the spike response of cells in the primary visual cortex (V1) is modulated by stimuli presented outside their RFs [1–7]. This surround interaction has important implications for signal integration, because it reflects a refinement of integration processes, with increased response selectivity [8–10]. However, the precise mechanism by which modulation of spike responses by surround stimuli leads to an increase of response selectivity is not completely understood.

A stimulus falling outside the RF, by definition, does not evoke a spike response, but it does robustly evoke synaptic potentials as revealed by intracellular [11, 12] or optical [13, 14] recording. If a stimulus is presented inside the RF while the subthreshold potential change evoked by the stimulus outside the RF persists, the cortical site representing the RF is expected to undergo response alteration in correlation with subthreshold potential changes produced by the surround stimulus. In the current study, we examined this aspect by focusing on the role of the subthreshold local field potential (sLFP) in surround interactions in V1 of the awake monkey. In order to examine the underlying interaction for non-homogeneous modulation that varies with location within the RF surround [15], we used Gabor patches to stimulate focal surround regions, rather than the annular stimuli that have been commonly used to examine surround interactions. Gabor stimuli evoke a smaller LFP response than annulus stimuli [16], which may be disadvantageous for studying surround interaction, but reveal specific spatiotemporal interactions [10]. We sequentially presented two identical Gabor stimuli with variable stimulus onset asynchrony (SOA), the first (S1) outside the RF evoking a sLFP change, and the second (S2) over the RF generating a spiking response. Varying SOA enabled the S2-evoked spiking activity to collide with a different phase of the S1-evoked sLFP. In previous studies of the relationship between spike activity and spike-triggered LFPs [17, 18] or stimulus-triggered LFPs [19, 20], suprathreshold LFPs were analyzed and the temporal relation between spikes and LFP was not manipulated. In contrast, we characterized the sLFP and examined its role in modulation of spike activity, while keeping S1 and S2 constant and only varying SOA. We found that the magnitudes of LFP and spike modulation varied across SOA, and were correlated, and that the time course of covariation between LFP amplitude and spike activity suggests a role of sLFP for spike modulation.

## Materials and Methods

### Animal preparation

#### Ethics statement

Two adult male monkeys (monkey *CR*:*Maccaca mulatta*, 7.5 years old; monkey *IR*: *Maccaca fascicularis*, 8 years old) were used. The experimental procedures were approved by the Seoul National University Institutional Animal Care and Use Committee (Permit Number:SNU-140408-7-1), and were in compliance with the U.S. National Institutes of Health guidelines. Incorporated ethical standards include an environmental enrichment program, such as routine contacts with other animals and expanded cage. Also included were regular veterinary care and tests provided by a dedicated personnel, and pharmacological aid ameliorating suffering associated with surgical procedures. These animals were housed in a colony maintained at a constant temperature and humidity and circulated with HEPA filtered-air. The animals were fed twice a day with sterile primate diet (Harlan Lab, USA) supplemented with bananas and apples. None of these animals were sacrificed for completion of the current study.

### Experimental procedures

Unless stated otherwise, the procedures employed in the current study for surgical procedures, experimental setup, spike sorting, and off-line analyses were identical to those described previously [21], as was the stimulus presentation paradigm [10]. Eye position was monitored with the scleral search coil method [22] or a camera (ET-49, 230 Hz, Thomas Recording, Germany). Each animal, after training, underwent three recording sessions a week, on average, over a period of three to five months.

We recorded extracellular potentials from the awake monkey primary visual cortex (V1) using platinum-iridium microelectrodes insulated with quartz with their impedance ranging between 1 and 4 MΩ at 1 kHz (Thomas Recording, Germany). For each recording session, the electrode and guide tube assembly was advanced until it touched the dura, and then the electrode was further advanced by a 5-CH minidrive (Thomas Recording, Germany), penetrating the dura. If one electrode failed to find a single cell, the next electrode was advanced. In order to reduce tissue drag during electrode penetration, care was taken to regularly thin the dura prior to recording session. The signal ground was the guide tube touching the dura. During recording sessions, the animal was seated on a monkey chair with its head restrained. Neural signals from the electrode were amplified by a preamplifier with a gain of 20 (Thomas Recording, Germany), and bifurcated to the main amplifiers for spike and local field potential (LFP) signals. The spike and LFP signals were amplified with bandwidths of 0.5 to 4 kHz and 0.1–140 Hz, respectively (Thomas Recording, Germany), digitized at a rate of 25 kHz with a 16-bit resolution (PCI-6052E, National Instruments), and stored. The LFP signals were later downsampled to 1 kHz for analyses.

After a cell was isolated, we first estimated its receptive field position with stimuli guided by a hand-operated computer mouse. Once the position was approximated, an optimal Gabor stimulus for the cell was quantitatively determined while the animal was fixating on a target. In order to isolate a single cell on an on-line basis, waveforms from neural activity were extracted and sorted based on peak-to-peak duration and amplitude of action potentials. For the isolated single cell, we sequentially estimated the orientation, horizontal and vertical positions, and size of the Gabor stimulus that evoked maximal activity. For estimating the optimal value in each of these dimensions, we randomly varied one dimension while keeping the other dimensions constant and averaged the number of spikes during post-stimulus time period between 50 and 200 ms over 5 to 10 repeated trials. Average spike counts against each stimulus dimension were fitted with a difference-of-Gaussian function. The optimal Gabor size was quantitatively determined with a spatial summation test [7] in which Gabor stimuli ranging in diameter from 0.2 to 2.0° in steps of 0.1 or 0.2° were randomized and presented at the center of the RF. The diameter of the Gabor stimulus that was associated with the maximum activity was taken as optimal size. For some cells, a diameter slightly larger than this was taken to prevent the surround stimulus from encroaching on the RF, because the Gabor stimulus defined the RF boundary. In most experiments, the chosen diameter was 1.6°. When the neural response did not saturate with an increase in stimulus diameter, which was rare, the largest tested stimulus (2.0°) was taken as optimal size. The RF size determined with such a spatial summation test yields relatively larger estimates than that estimated with stimuli eliciting minimal responses [7, 23, 24] or reverse correlation methods [8]. However, an S1 immediately adjacent to the S2 occasionally evoked a spike response even when the RF was determined this way, indicating that our estimate of RF size was not large enough in those cases. This motivated us to further separate the S1 from S2 in later phases of experiments, from one to 1.5 diameters of RF, center to center.

**Fig 1:**
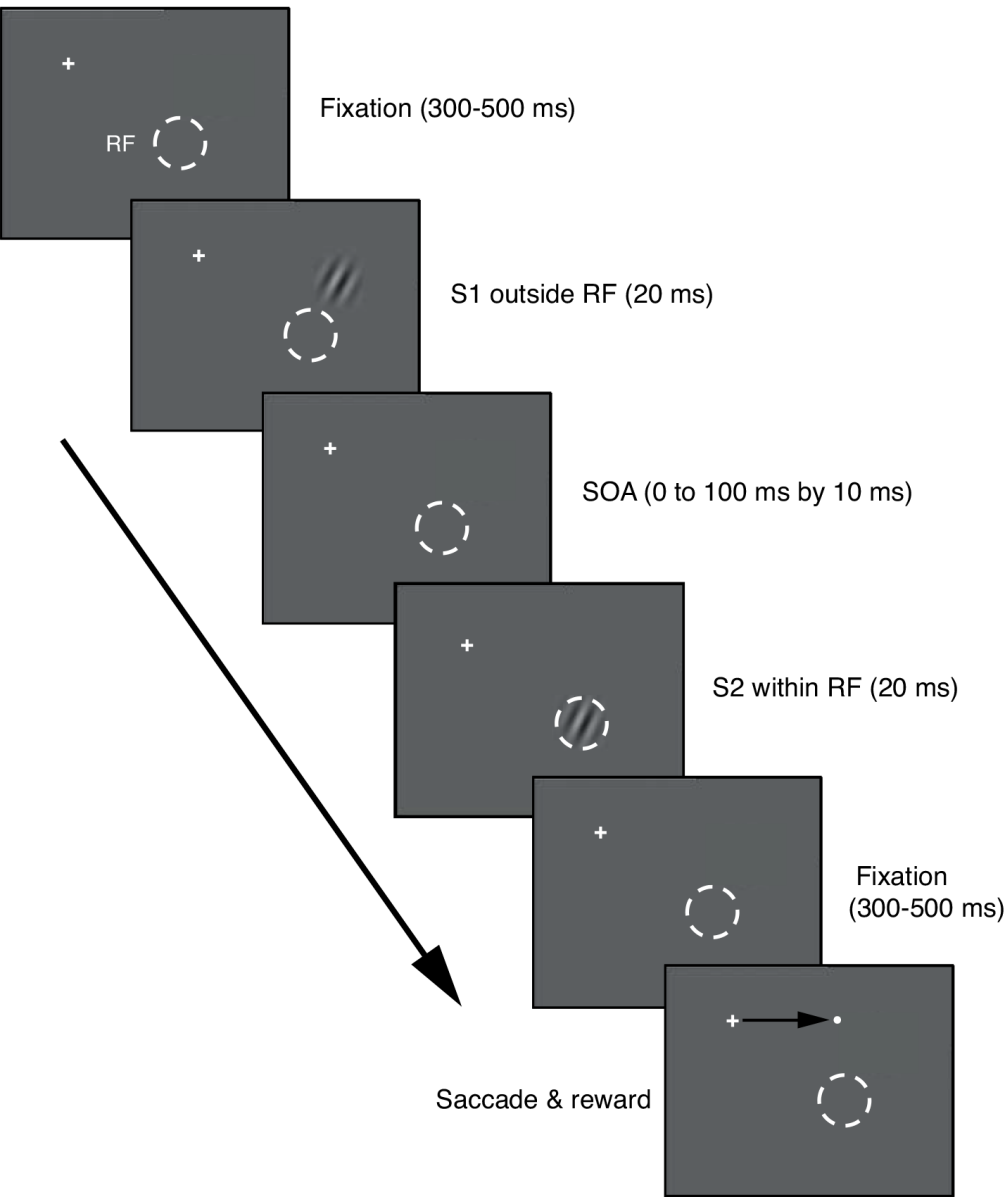
Trial structure for S1-S2 sequence stimuli. A white cross indicates central fixation and a dashed white circle (invisible to the animal) represents the boundary of the classical receptive field (RF). The stimulus onset asynchrony (SOA) between S1 and S2 varied in steps of 10 ms. In some trials, only S1 or S2 was presented. The SOA of 0 ms corresponds to simultaneous presentation of S1 and S2. After completion of S1-S2 presentation, a saccade target was presented at one of four randomly-chosen locations, up, down, left, and right with respect to the fixation target. These target positions never were in the RF.

Once the optimal Gabor stimulus for a cell was determined, the animal performed the task of main experimental paradigm (Fig 1). Each trial began with onset of a fixation target (0.3°x0.3°, red dot) at the center of a gamma-corrected 24” flat CRT monitor (Sony GDM-FW900, 800x600, 100 Hz) that was controlled by computer programs written in Matlab (The Mathworks) using Psychophysics Toolbox [25, 26]. After the eye position entered a circular fixation window of 1.5° in diameter centered on the fixation target, the Gabor stimuli were presented after a variable delay period of 300–500 ms as explained below. In the trials with two stimuli (Fig 1), the first Gabor stimulus (S1) appeared at a location outside the RF, and then with an SOA ranging from 0 to 100 ms in steps of 10 ms, the second stimulus (S2), chosen to be optimal for the cell, appeared at the RF. The duration of each stimulus was 20 ms. The stimuli were presented on a gray background with a mean luminance of 8.65 cd/m^2^, a contrast of 64%, and a typical spatial frequency of 2 cycles/°. S1 was located eccentric to the RF, in line with the cell's preferred orientation. The orientations of S1 and S2 were collinear with the phase aligned, and they were identical except for their locations. In some trials, only S1 or S2 was presented. The S1-alone condition revealed the subthreshold LFP, and also served as a check on whether the stimulus was indeed outside the RF, based on the absence of an evoked spike response. The S2-alone condition served as a reference against which neural activity in response to the S1-S2 sequence could be evaluated. In order to examine the effects of distance between S1 and S2, we used multiple S1s in some experiments. For this, we varied the location of S1 in discrete steps along the axis collinear to the orientation of the stimulus, ranging from 1.6 to 10° from the center of RF (from 1.5 to 4.5 RF diameters or slightly larger in some cases), while maintaining the location of S2 at the RF. Thus, the trial types included 11 SOA conditions (0 to 100 by 10-ms step) multiplied by the number of S1 positions, plus S1- and S2-alone conditions. These trial conditions were pseudo-randomized within a block, and any trials aborted due to poor fixation were repeated at the end of each block. After the stimulus went off, a saccade target appeared at a location randomly chosen from four locations, acquisition of which was subsequently rewarded with drops of fruit juice. The saccade task was used to ensure animal’s vigilance.

### Data analysis

During off-line analysis, invalid trials were discarded. These included trials in which the eye position transgressed the circular fixation window of 1.5° diameter before completion of stimulus presentation, or the eye velocity exceeded 50°/s during the period of stimulus presentation. Trials with outlying firing rates (>3 SDs from the trial type mean) during the post-stimulus time period of 50–150 ms following S2 (throughout the paper, statements about post-stimulus timing always refer to S2 onset), as well as those with a stimulus duration other than intended (as ascertained with the output of a photometer facing the stimulus monitor), were also discarded.

Spike waveforms in valid trials were extracted and sorted off-line [21]. Based on the compiled spike sequences for each stimulus condition, a spike density function was derived with an asymmetric kernel function of a total length of 150 ms, R(t)=(1−e−tτg)∙(e−tτd), where *τ*_*g*_ and *τ*_*d*_ are time constants for 1 ms of growth and 20 ms of decay, respectively [27].

### Retinotopic map and cortical magnification factor

In order to evaluate the effects of distance between the S1 and S2 positions on the LFP change and spike response, the distance on the cortex between the sites of activity evoked by S1 and S2 was estimated as follows. A point on the stimulus monitor, (*x*,*y*), was first transformed into polar coordinates, $e=\sqrt{x^{2}{+y}^{2}}$, $a=\arctan\left(\frac{y}{x}\right)$ where *e* is eccentricity and *a* is inclination. The position of the activity evoked by the point on the cortex, (*X*,*Y*), was estimated with, X=λln(1+ee0), and *Y* = −*λae*/(*e*_0_ + *e*), where *λ* = 12 *and e*_0_ = 0.75 [28]. The distance between the cortical representations of S1 and S2, (*X*_*1*_,*Y*_*1*_) and (*X*_*2*_,*Y*_*2*_) was estimated as $d=\sqrt{{\left(X_{2}-X_{1}\right)}^{2}+{\left(Y_{2}-Y_{1}\right)}^{2}}$.

### Quantification of LFP

During off-line analysis, we applied a strict ‘subthreshold’ criterion to ensure that S1 was indeed outside the RF. For this, if S1 alone evoked a spike response larger than 5% of that evoked by S2 alone in terms of mean spike density during the post-stimulus period of 50–150 ms, we excluded those cells from further analysis. For the remaining cells, we isolated S1-evoked LFPs, that is, sLFPs, by averaging LFP traces aligned at S1 onset. Similarly, we isolated S2-evoked LFPs and S1-S2 sequence-evoked LFPs by aligning and averaging LFP traces at S2 onset. These procedures eliminate non-stimulus related LFPs [29].

The S1-evoked sLFPs showed distinct peaks whose latency varied depending on the S1 position. In order to estimate this latency, the positive and negative peaks were localized at zero-crossing points of the time-differentiated LFP signal after smoothing with a 50-ms moving average. The amplitude of the sLFP was taken from the change in potential between positive and negative peaks.

In order to determine the latency of S2-evoked LFP changes that were initially downward, we first smoothed and differentiated the mean LFP of each type with a 20-ms moving average. The start of a downward change in the raw LFP after stimulus onset, as given by the time when the first time-derivative of the mean LFP crossed a threshold (1.75 μV/s) away from the baseline level (mean baseline across recoding sites was 0.04 μV/s), was taken as the latency of S2-evoked LFP.

In order to quantify the response magnitude of LFP for each stimulus condition, we calculated the root mean square (RMS) magnitude from the baseline-subtracted, stimulus-evoked LFP during the post-stimulus period of 0–300 ms:

$$RMS=\sqrt{\frac{\sum_{i=n}{\left(x_{i}-\bar{x_{b}}\right)}^{2}}{n}},$$

where *x*_*i*_ is the LFP at the *i* ^*th*^ time bin, $\bar{x_{b}}$ is the baseline LFP averaged during the period from -200 to 0 ms of stimulus onset, and *n* is the total number of time bins, 300 in this case. The baseline level was subtracted to prevent trial-to-trial variability of the overall LFP level from affecting the response magnitude. In order to obtain an estimate of the stimulus-evoked LFP magnitude change, the RMS percentage change was calculated as follows,

$$RMS\;\%\;change=\frac{{RMS}_{S1-S2\;Sequence}}{{RMS}_{S2-alone}}\times 100,$$

where *RMS*_*S1-S2 Sequence*_ is the RMS from the S1-S2 sequence condition, and *RMS*_*S2-alone*_ is the RMS from the S2-alone condition. We calculated the RMS percentage change for each trial and derived a geometric mean across trials.

### Correlation between spike and LFP modulation

In order to determine the time course by which SOA-dependency of spike activity matches SOA-dependency of the LFP, we calculated the instantaneous Pearson product-moment correlation coefficient, *c(t)* in 1-ms steps as follows:

$$c(t)=\frac{\sum_{i=1}^{11}\left[r_{i}-\bar{r}][v_{i}(t)-\bar{v(t)}\right]}{\sqrt{\sum_{i=1}^{11}{\left[r_{i}-\bar{r}\right]}^{2}}\sqrt{\sum_{i=1}^{11}{\left[v_{i}(t)-\bar{v(t)}\right]}^{2}}}$$

where *r*_*i*_ is the mean firing rate during the post-stimulus time period of 50–150 ms obtained for the *i* ^*th*^ of 11 SOA conditions, *v*_*i*_(*t*) is the instantaneous LFP at time *t* for the corresponding *i* ^*th*^ SOA condition, and $\bar{r}$ and $\bar{v(t)}$ are their respective means. The correlation timecourse was calculated for each cell, and within each cell the maximum and minimum coefficients and their times were determined. The significance of the maximum and minimum coefficients was statistically tested with a bootstrap method, in which the obtained correlation was tested against the probability distribution of the correlation coefficient for the corresponding time derived from 1000 simulations of randomized shuffling across SOA conditions. Basically, this procedure temporally localized the pattern of SOA-dependent spike activity in the LFP data, and enabled us to determine the temporal precedence between the SOA-dependent spike activity and the LFP change, and, thus, to determine if the data are consistent with a modulatory role of sLFP.

### Control of eye position

A critical issue for comparing the response magnitude across SOA conditions is the stability of eye position, because trained monkeys can make short-latency saccades to visual targets, and the latency can be close to 100 ms [30] or even less [31]. We examined eye stability in three ways. First, for each SOA condition, we checked whether the mean horizontal and vertical eye positions during the 20-ms presentation of S2 were within 2 or 3 SDs of the mean of the site. Second, we determined if the presentation of S1 resulted in a change in eye position by the time S2 was presented by examining the statistical difference in the mean radial eye positions, $\sqrt{h^{2}+v^{2}}$, where *h* and *v* are horizontal and vertical eye positions, respectively, between the S2-alone condition and the S1-S2 sequence condition with an SOA of 100 ms. The reason for using the SOA of 100 ms for this comparison was that if there had been any change in eye position, it could have most likely occurred following the preceding S1. We examined all the mean eye positions for each SOA conditions for 595 stimulus conditions tested at 31 cortical sites: 31 S2-alone, 47 S1-alone (30 near and 17 farther S1), and 517 S1-S2 sequence (47x11 SOA). In 581 of 595 conditions, the mean horizontal and vertical eye positions were within 2 SD of the mean of the site, and in the remaining 13 conditions, the eye position (either horizontal or vertical) was within 3 SD of the mean, indicating that stable eye position was maintained during S2 presentation. The mean radial eye position during the S2-alone condition and the S1-S2 sequence condition with an SOA of 100 ms (31 S2-alone and 47 S1-S2 conditions) did not differ significantly (p = 0.12, two-sample t-test).

Finally, in order to examine whether occurrence of microsaccades is related to SOA, we examined the number of microsaccades for all trials from 19 of 30 cells for which nearest S1 was tested and microsaccades were relatively unambiguously detected. To detect microsaccades during fixation, initial candidates were localized with a velocity criterion (>15 deg/s) and the onset and offset of microsaccades were determined with an acceleration criterion (550 deg/s^2^, S1C–S1E Fig) [32] with visual inspection. Overall, in 1510 of 3638 trials 1612 velocity peaks corresponding to candidate microsaccades were detected within the analysis window of 300ms starting from -100ms to 200ms of S1 onset. Of these,1524 were taken as microsaccades, excluding those with outlying amplitudes beyond 2 SDs of the mean. The mean amplitude of the 1524 microsaccades was 18.78 (±14.16) min arc. The overall mean rate of microsaccade occurrence during the analysis window was 1.44 (±0.10) microsaccades/s. These values of the amplitude and rate of detected microsaccades are close to those obtained in the previous study [32]. For the SOA conditions of 0 through 100ms, the mean rates (SD) were 1.40 (±0.44), 1.27 (±0.67), 1.33 (±0.51), 1.45 (±0.42), 1.42 (±0.53), 1.49 (±0.74), 1.40 (±0.59), 1.49 (±0.56), 1.59 (±0.41), 1.38 (±0.61), and 1.58 (±0.85) microsaccades/s, respectively (S1F Fig). None of these were significantly different from the overall mean occurrence (t-test, p>0.10 for all comparisons).Thus, we conclude that the eye position was not significantly different across SOA conditions.

## Results

### Data summary

The complete spike and LFP data described in this report are based on 62 single cells recorded from 62 sites in the dorsal operculum of V1 during 62 recording sessions in two awake monkeys. During off-line analysis, it was found that in 22 of these sites, S1 encroached upon the RF that had been defined with the spatial summation test, so that the ‘subthreshold’ criterion (spike response larger than 5% of that evoked by S2 alone)was violated. Accordingly, the data from these 22 sites were excluded from those analyses that required strict surround criteria. The mean diameter of the RF (and thus the size of S2) of the remaining 40 valid sites was 1.6 ±0.03°. The mean eccentricities of RFs for these sites, 25 from monkey *CR* and 15 from monkey IR, were 4.69 ±1.03° and 4.04 ±0.34°, and the mean recording depths from the surface of the dura were 1.39 ±0.34 mm and 0.91 ±0.32 mm, respectively.

### Properties of subthreshold LFP

**Fig 2:**
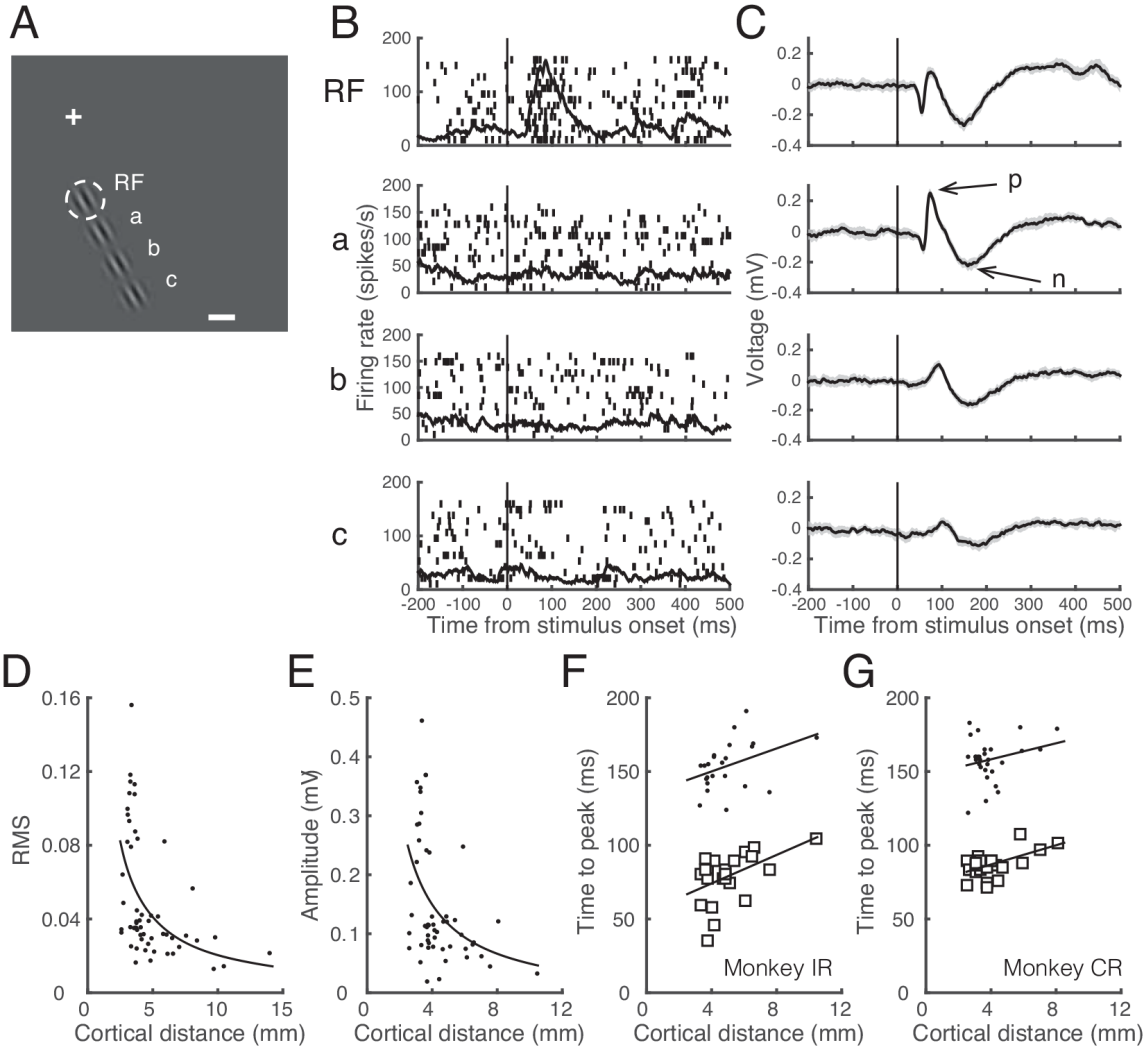
Properties of subthreshold (sLFP). **A.** Spatial layout of stimulus configuration: the cross marks the central fixation target; dashed white circle represents the RF (1.6° in diameter, centered at 0.6° right and 4.1° down); a Gabor stimulus at the RF and three identical stimuli positioned outside the RF spaced at intervals of one RF diameter away from the RF along the direction collinear to preferred orientation (a, b, and c); the calibration bar indicates 1°. The distance of stimuli at a, b, and c from the RF center in cortical dimension was estimated to be 3.28, 5.89, and 8.06 mm, respectively. **B, C.** Raster and spike density plots (B) and mean LFP traces (C). Shading in C indicates ±2 SE. From top to bottom, responses to the Gabor stimulus at the RF alone, and those at a, b, and c alone are shown, aligned at the stimulus onset times (vertical lines). Note that a robust LFP change was evoked by the stimuli at a, b, and c, while the spike activity remained unchanged. The dominant positive and negative peaks of this sLFP are indicated as ‘p’ and ‘n’, respectively. **D.** Power (RMS) of sLFP as a function of cortical distance between the center of Gabor stimulus in the RF surround and the center of RF, extracted from data on 56 surround stimuli tested at 40 cortical sites. The curve is a fitted function in the form of $y=A\frac{1}{x}+B$, following the inverse distance law of sound pressure, where *x* is cortical distance in mm between the center of Gabor stimulus in the RF surround and the center of RF. Parameter *A* was estimated to be 0.21, and its 95% confidence limits were 0.10 and 0.31; *B* was estimated to be 0.00. **E.** The amplitude of the sLFP, as measured from positive to negative peaks (as shown in C) as a function of cortical distance between the center of Gabor stimulus in the RF surround and the center of RF for 52 conditions. Four of the 56 conditions in D, for which the peaks could not be determined, were excluded. The curve is a fitted function in the same form as in D. Parameter *A* was estimated to be 0.67, its 95% confidence limits were 0.27 and 1.07; *B* was estimated to be 0.02. **F, G.** Latency to positive (open squares) and negative (dots) peaks of sLFP as a function of cortical distance between the center of Gabor stimulus in the RF surround and that at the center of RF, separately for monkey IR (F) and monkey CR (G) from the 52 conditions shown in E. The data were separately fitted with linear regression equations: *y* = 4.83*x*+54.79 and *y* = 3.90*x*+134.46 for monkey IR, and *y* = 3.27*x*+73.90 and *y* = 2.71*x*+147.44 for monkey CR, for latency to the positive and negative peak, respectively, where *x* is cortical distance between the two stimuli (p<0.05 for all cases).

In order to characterize sLFP, we paid particular attention to spike responses evoked by S1. Fig 2A–2C illustrates the activity of a representative cell for which the effects of presenting a Gabor stimulus at each of three locations in the RF surround (a-c, Fig 2A) was tested. Although the Gabor stimuli in the RF surround did not evoke spike response (Fig 2B, a-c), it evoked changes in LFP consisting of positive and ensuing negative peaks of varying magnitudes (Fig 2C). We refer to this LFP change evoked by the surround stimulus alone without a criterion spike response (see Materials and Methods) as a *subthreshold LFP*, or *sLFP*, throughout the text. Fig 2D–2G provides quantitative summaries of the dependency of sLFP on the spatial distance between the center of the Gabor stimulus in the RF surround and the center of RF. As the distance between the two in the cortical dimension increased, the power of sLFP (Fig 2D) and the positive-to-negative peak amplitude of sLFP (Fig 2E) decreased. Similarly, as the distance between the two increased, the temporal interval between the onset of stimulus in the RF surround and the time of positive or ensuing negative peak of the sLFP increased (Fig 2F and 2G). The sLFP change was evoked by the surround stimuli that were represented at cortical distances up to 10 mm away. These patterns of LFP change were similar between both monkeys. Note that the signal ground was the guide tube above the dura, and the distance between the electrode and guide tube was less than the cortical thickness, and thus the sLFP is presumed to be local.

From the latency-distance relationship of Fig 2F and 2G, the propagation speed of sLFP can be estimated. The inverse of the slope of the regression line, an estimate of propagation speed, was 0.21 and 0.26 m/s in monkey IR (Fig 2F) and 0.31 and 0.37 m/s in monkey CR (Fig 2G), for positive and negative peaks, respectively. These estimates agree well with previous estimates of propagation speed through horizontal connections, which range between 0.05 and 0.5m/s [11, 13, 17, 19, 33, 34]. Note that for an angular distance of 1.6° between the Gabor stimuli in the RF center and the most adjacent surround (a typical RF diameter in this study) or for its corresponding distance of 2.58 to 4.87 mm (mean = 3.63 mm) in cortical dimension depending on eccentricities examined in the current study, the transmission speed between the sequential stimulus sites corresponds to 16–160°/s for SOAs of 10–100 ms. We verified that this speed induced a perception of apparent motion, consistent with previous report [35]. This speed is comparable to the velocity tuning of MT neurons [36, 37] and to the motion velocity eliciting propagating waves in V1 [38], suggesting a role of sLFP for processing of global motion that extends both inside and outside the RF.

### sLFP and modulation of spike response

**Fig 3:**
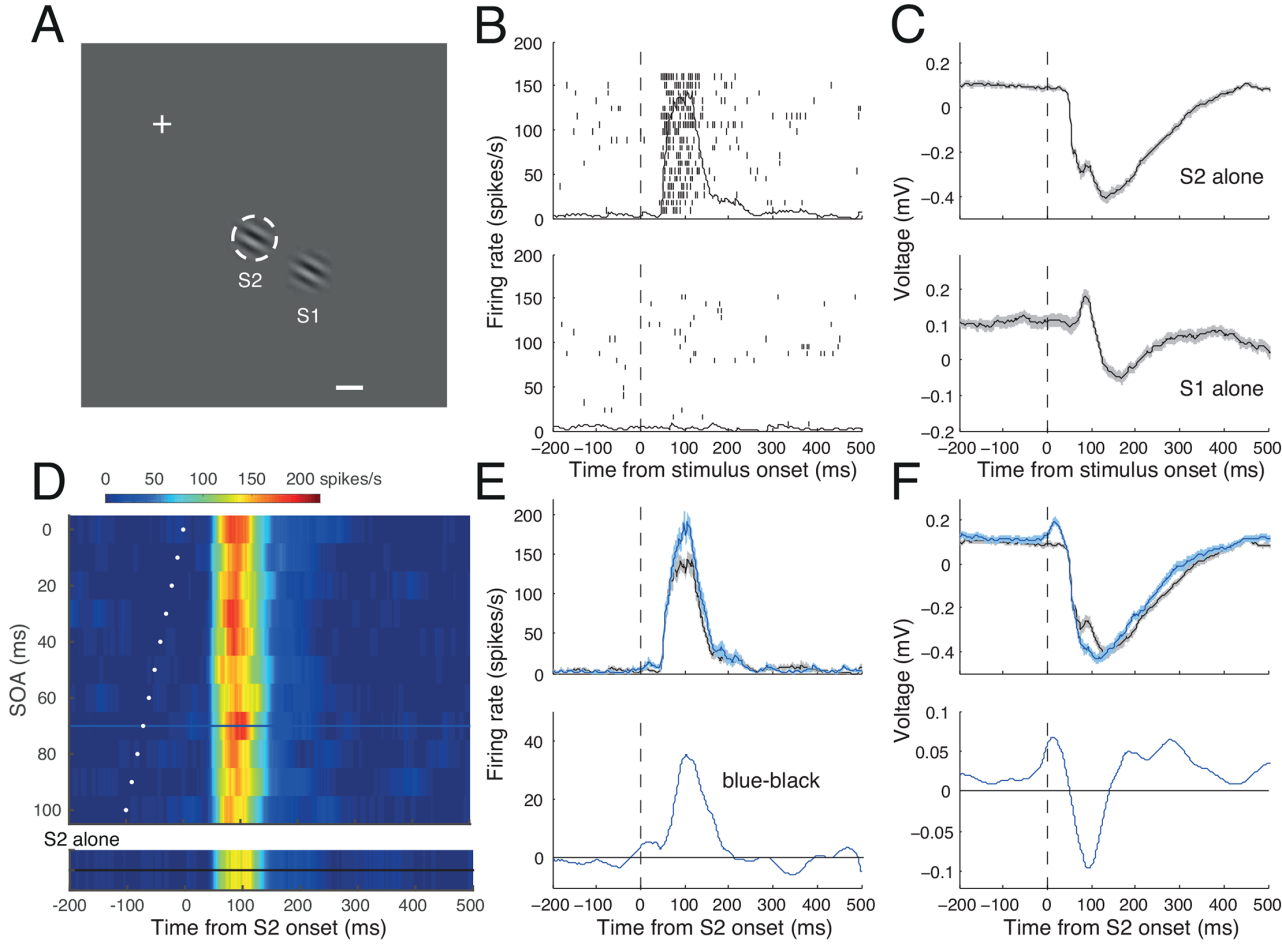
Spike and LFP activity of a representative cell. **A**. Spatial layout of stimulus configuration: the cross marks the fixation target; the dashed white circle(invisible to the animal) shows the boundary of the RF; the calibration bar indicates 1°. The RF was centered 3.3° right and 4.2° down. Two Gabor stimuli, one at the RF (S2), and the other in the RF surround (S1) are shown. **B, C**. Raster and spike density plots (B) and mean LFP traces (C) in response to S2 alone (upper) and S1 alone (lower), aligned at their onset times (dashed vertical line). Shadings in C indicate ±2 SE. Y-axis indicates spike density in spikes/s in B, and LFP amplitude in mV in C. Note that a robust LFP change was recorded in response to S1 alone, while the cell did not discharge spikes. **D**. SOA time plot for response modulation during trials with S1-S2 sequence stimuli, showing spike activity as a function of SOA and time, aligned at S2 onset. Activity is coded by color, as indicated by the calibration bar at top. White dots indicate the time of S1 onset for each SOA condition. The spike density for the S2-alone condition is given in a separate color map at bottom for comparison. Note that depending on SOA, spike density varied considerably in terms of magnitude and time course. Spike density for an SOA of 70 ms is indicated by the blue horizontal line, whereas the reference density for S2 alone (at bottom) is indicated by the horizontal black line; the time courses of both are shown in the upper panel of E with the same color coding. **E, F.** Upper: Spike (E) and LFP activity (F) in response to S1-S2 sequence stimuli with an SOA of 70 ms (blue), aligned at the time of S2 onset (dashed vertical lines). In each panel, a black trace indicates the reference of the S2-alone condition. Shadings indicate ±2 SE. Lower: The magnitude of modulation (S1-S2 sequence minus S2-alone) in firing rate (E) or LFP (F) is plotted for the SOA of 70 ms.

Fig 3 illustrates the results from an example cell for which S1 was presented 1.5 RF diameters away from the RF (Fig 3A). The stimulus at the RF (S2) evoked a burst of spike activity (Fig 3B, upper) accompanied by a simultaneous change in LFP (Fig 3C, upper). In contrast, the S1 did not evoke a spike response (Fig 3B, lower), but it robustly evoked an LFP change with a distinct positive peak followed by a negative peak (Fig 3C, lower). When S1-S2 sequence stimuli were presented, modulation of spike activity was apparent depending on SOA (Fig 3D). For example, compared to the reference activity in response to S2 alone (Fig 3D, bottom; black trace of Fig 3E), the spike response increased for an SOA of 70 ms (blue traces in Fig 3D and 3E). For an SOA of 70 ms, S1 induced a positive change in the LFP before the start of a negative response, resulting in a pronounced negative peak (Fig 3F, lower panel). Changes in spike and LFP responses induced by S1 at 70-ms SOA (blue traces) occurred at specific times (Fig 3E and 3F, lower panels).

**Fig 4:**
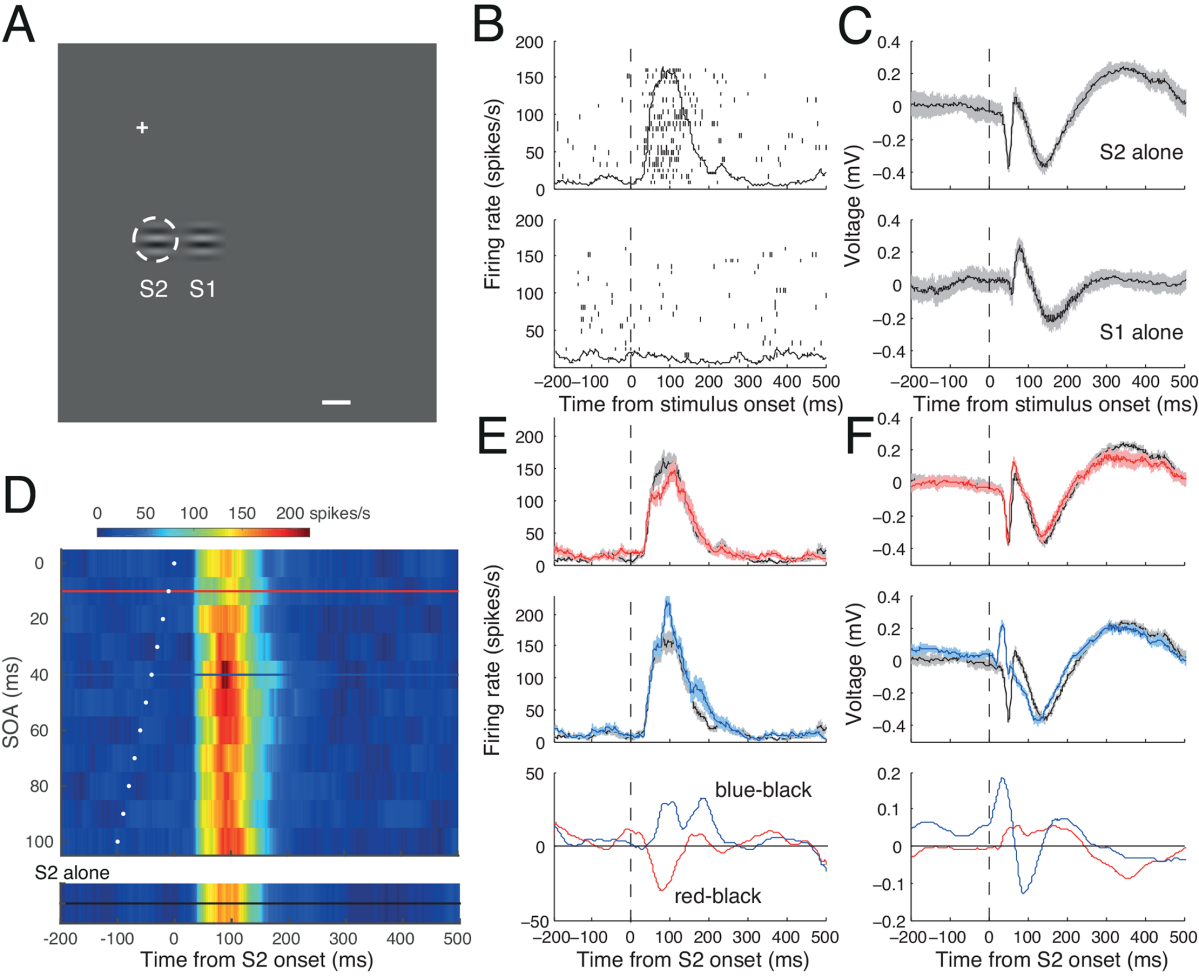
Spike and LFP activity of another representative cell. Same conventions as Fig 3. **A**. The RF was centered 0.6° right and 4.0° down. **B, C**. Raster and spike density plots (B) and mean LFP traces (C) in response to S2 alone (upper) and S1 alone (lower) aligned at their onset times. Shadings indicate ±2 SE. **D**. SOA time plot. Representative spike densities for SOA of 10 and 40 ms are indicated by red and blue horizontal lines, respectively. **E, F.** Upper and middle panels: Spike (E) and LFP activity (F) in response to S1-S2 sequences with SOAs of 10 (red) and 40 ms (blue); black traces indicate references taken from the S2-alone condition. Bottom panels: The magnitude of modulation (S1-S2 sequence minus S2-alone condition) in spike (E) and LFP (F) are plotted for SOAs of 10 ms (red) 40 ms (blue). Note that for the SOA of 10 ms, the magnitude of modulation in spike activity was negative (suppressed) and the modulation of LFP was relatively weak, whereas for the SOA of 40 ms, the magnitude of modulation in spike activity was positive (facilitated) and the modulation of LFP was relatively strong.

Fig 4 illustrates another example cell for which S1 was tested at one RF diameter away from the RF (Fig 4A). Again, S2 evoked a burst of spike activity (Fig 4B, upper) and a simultaneous change in LFP (Fig 4C, upper). The S1 stimulus did not evoke a spike response (Fig 4B, lower), but it robustly evoked an sLFP (Fig 4C, lower). Modulation of spike activity by S1 was apparent depending on SOA and time (Fig 4D). For example, the spike response decreased for the SOA of 10 ms (red traces in Fig 4D and 4E), and increased for the SOA of 40 ms (blue traces in Fig 4D and 4E). Note again that spike modulation did not occur for the entire spike response, but rather was confined within fixed temporal windows. For the SOA of 10 ms, the spike response decreased only during an interval about 100 ms after target onset, but the spike response outside this interval remained unchanged (Fig 4E, up and bottom panels), and for the SOA of 40 ms, the change occurred in two intervals for this cell (Fig 4E, middle and bottom). Corresponding mean LFP traces are shown in matching colors in Fig 4F. For the SOA of 10 ms, the LFP change caused by S1 was subtle, only slightly modifying the later phase of the LFP response (Fig 4F, up and bottom), whereas for the SOA of 40 ms, S1 added an LFP change at a very early phase, nullifying the negative and subsequent positive peaks of the S2-alone condition (Fig 4F, middle and bottom).

The S1 effects were mostly facilitative for the cell of Fig 3 (Fig 3D), whereas they were both suppressive and facilitative for the cell of Fig 4 depending on SOA (Fig 4D). Suppressive effects by surround stimuli have been dominant in the literature (for reviews, see [39–41]), but both suppression and facilitation of spike activity have been noted with natural stimuli [42], and with focal Gabor stimuli identical to those used in the current study [10]. Below we summarize the modulatory effects of S1 across SOA.

### Spike and LFP modulation and effects of SOA

**Fig 5:**
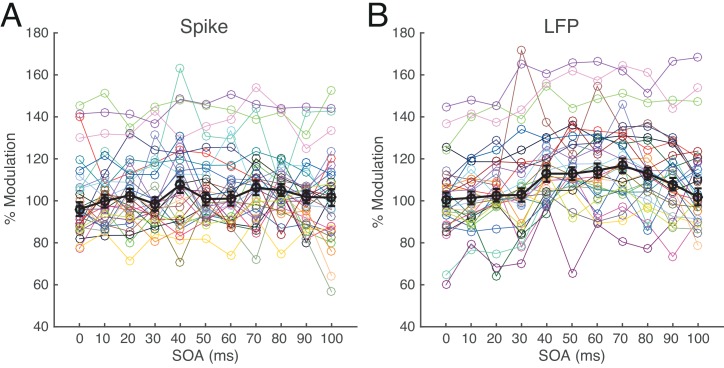
Effects of S1 on spike (A) and LFP (B) response across 11 SOA conditions. Each colored symbol represents the mean magnitude of spike (A) or LFP (B) response in percentage with respect to S2-alone condition for corresponding SOA condition of each of 30 cells for which nearest S1 was tested. Black symbols represent median values of those means with 1SEs. Percent modulations less than 100 indicate suppression and those larger than 100 indicate facilitation by addition of S1.

The magnitude of spike modulation was taken from the percentage change in spike density in the 50–150 ms period following the S2 onset during S1-S2 sequence conditions relative to that during S2-alone condition. The magnitude of LFP modulation was similarly quantified by the percentage change in RMS power on a trial-by-trial basis (see Materials and Methods). In 9 of the 40 valid cells, only S1-alone and S2-alone conditions were tested. From 30 of the remaining 31 cells, we selected the S1-S2 sequence stimuli for the S1 closest to the RF (for one site, the closest S1 was not tested, and for some sites, multiple S1 positions were tested), and calculated the magnitude of spike and LFP modulation for each SOA condition from these sequence conditions. The spike and LFP responses during S1-S2 sequence conditions showed variable modulation compared to S2-alone condition (Fig 5). Overall, suppressive (46.06%) and facilitative (53.94%) modulations similarly observed for spike activity (Fig 5A), whereas facilitative modulation (71.21%) was more frequent than for suppressive (28.79%) for LFP (Fig 5B). The modulation varied across cells and SOA; for some cells, facilitation or suppression was dominant for most SOAs, and for others, modulation was pronounced for one or more SOAs.

**Fig 6:**
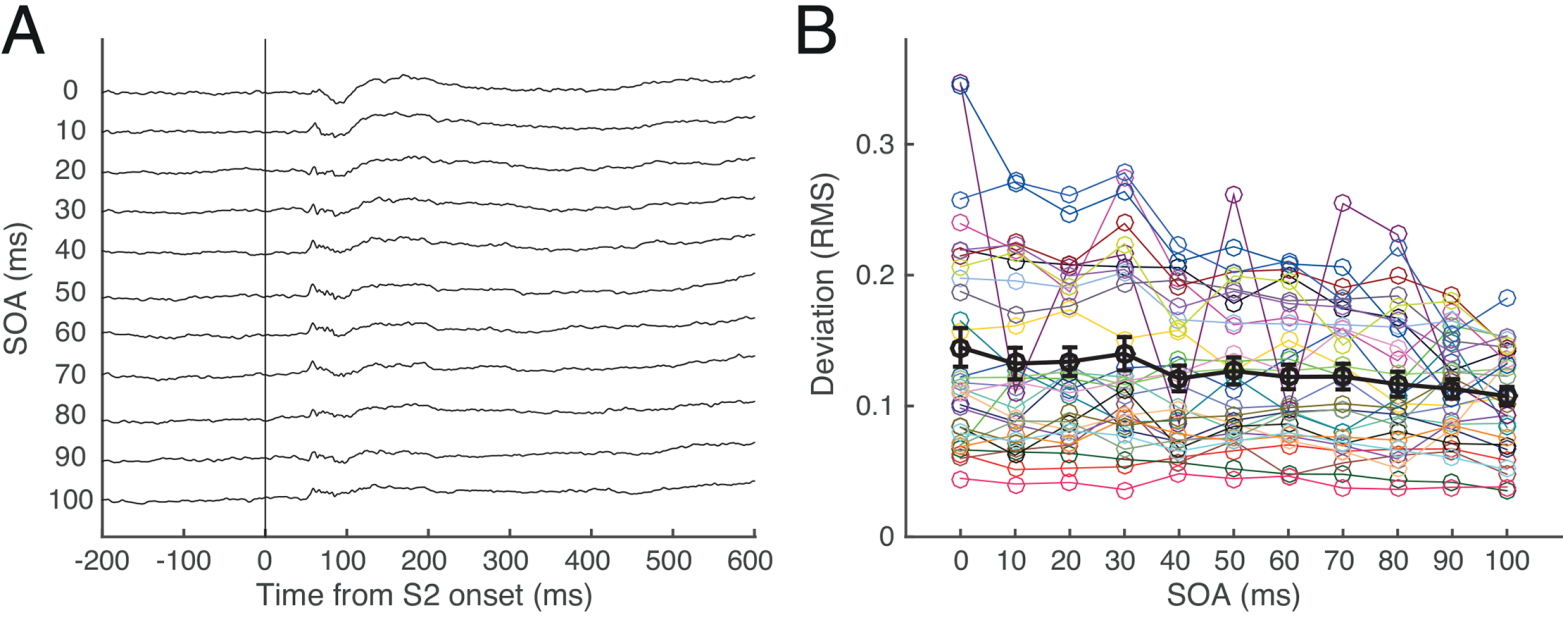
Deviation of LFP from linear sum in the representative cell of Fig 3. **A.** Shown are LFP traces in an arbitrary unit for each SOA condition derived by the mean LFP traces observed during S1-S2 sequence stimulation minus the SOA-adjusted linear sum of S1-evoked LFP and S2-evoked LFP. **B.** Deviation of LFP in RMS power. Each colored symbol represents the mean deviation across SOA conditions for each of 30 cells shown in Fig 5. Black symbols represent their mean values with 1SEs.

During S1-S2 sequence condition, S1-evoked sLFP and S2-evoked LFP are thought to interact with each other. The observed LFP during S1-S2 sequence condition deviated from the SOA-adjusted linear sum of S1-evoked LFP and S2-evoked LFP. The deviation occurred mostly during S2-evoked LFP response period (Fig 6A) and the deviation magnitude was greater for shorter SOAs (Fig 6B). The magnitude of this deviation appears not related with spike or LFP modulation, as can be seen in Fig 5 where percentage modulation did not decrease with SOA.

### Correlation between LFP and spike activity

**Fig 7:**
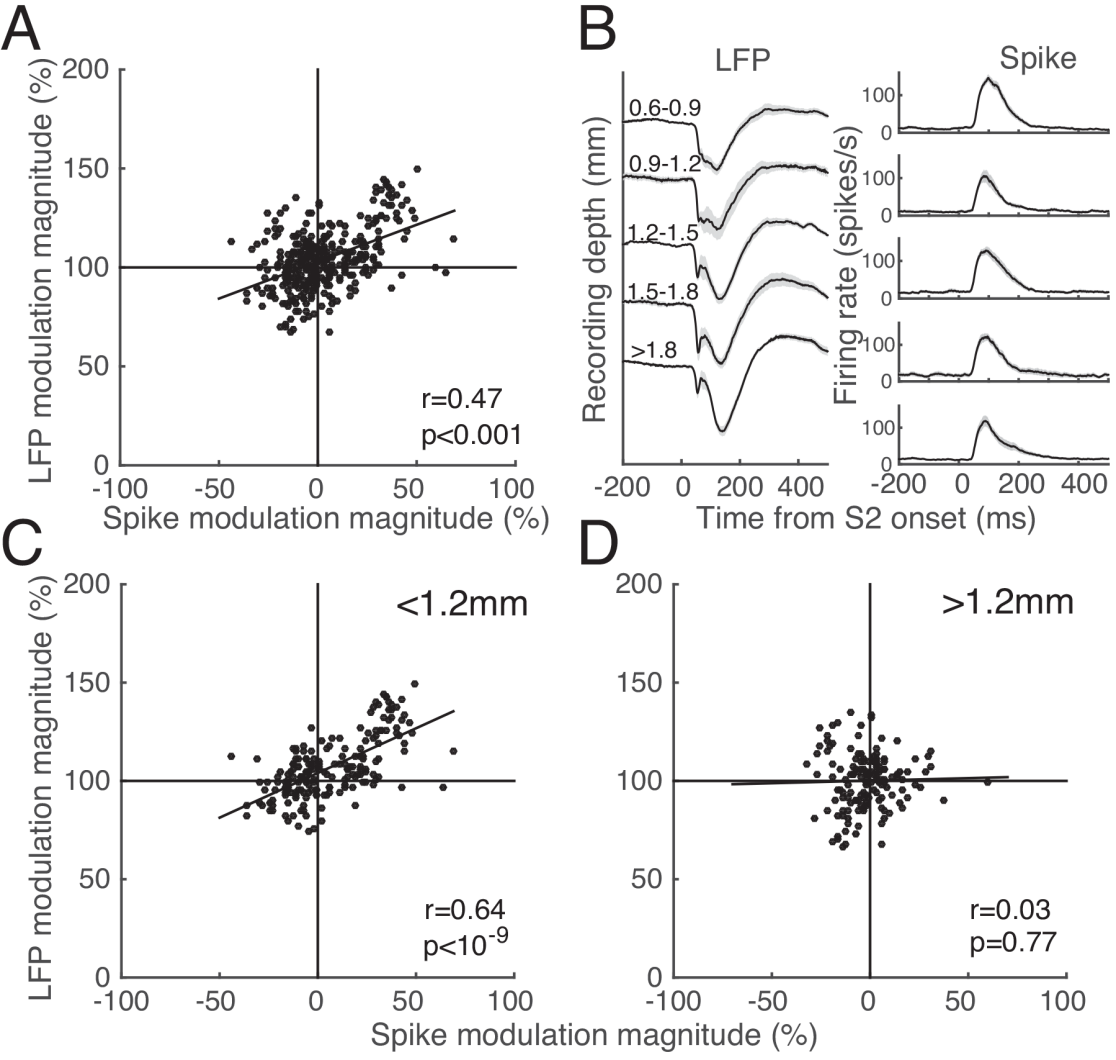
Relationship between spike and LFP modulation. **A.** Scatter plot showing the percentage changes in spike activity and RMS LFP power in the S1-S2 sequence relative to the S2-alone condition for 330 stimulus conditions (11 SOA conditions X 30 sites). They are positively related as indicated by the Pearson correlation coefficient and its p-value inside the panel. **B.** LFP (left) and corresponding spike density (right) traces from all 62 sites in which S2 alone was tested, averaged for five depth groups divided into depth segments of 300μm, measured from the surface of the dura. The deepest trace (bottom) includes all recording sites below 1800μm from the dura. The shading represents ±1 SE. **C, D.** Relationship between spike and LFP modulation subdivided into two depth groups, upper (C, <1.2mm) and lower (D, >1.2mm). Same convention as A.

The modulation of LFP response in the S1-S2 sequence condition was likely contributed by the sLFP evoked by S1. In order to reveal the roles of sLFP for spike modulation, we first determined whether the modulation of spike activity by S1 was quantitatively related to that of LFP. Fig 7A illustrates the percentage changes in spike activity against RMS LFP power in the S1-S2 sequence relative to the S2-alone condition for these 30 sites. They were positively correlated (r = 0.47, p<0.001).

It is known that the stimulus-evoked LFP change varies across cortical depth [43, 44], and that the pattern of surround interaction differs across cortical layers[45]. In our data, the pattern of LFP evoked by S2 changed gradually with the recording depth, and a negative peak additionally appeared for relatively deeper recording sites, although the spike activity evoked by S2 remained unchanged (Fig 7B). Although our recording was made with a single electrode, this appeared to be consistent with additional stimulus-locked negative potentials recorded from lower layers in monkey V1 [46, 47] and A1 [48]. We reevaluated the 30 sites described above after subdividing them into two depth groups, upper (<1.2 mm) and lower (>1.2 mm). Note that the recording depth was taken as the distance the electrode was advanced at right angles from its touchdown on the surface of the dura, and this was subject to considerable error due to uncontrolled tissue drag during penetration of the dura, despite routine thinning. A few relatively large depths of about 3 mm are undoubtedly due to such errors. Nevertheless, we observed that the correlation between the percentage changes in spike activity and LFP RMS power relative to the S2-alone condition was statistically significant in 17 upper group cells (Fig 7C), but not in 13 lower group cells (Fig 7D) (r = 0.64 vs 0.03). This was consistent in both animals; in monkey CR, the correlation between spike and LFP modulation was 0.64 for 4 upper group cells, and 0.05 for 13 lower group cells, and in monkey IR, it was 0.78 for 7 upper group cells, and 0.40 for 6 lower groups cells.

We note additional differences between these two depth groups. First, the magnitude of spike modulation differed; facilitative spike modulation (>0%) occurred more frequently in the upper group (62.81%, 76 out of 121 SOA conditions) than in the lower (42.23%, 87 out of 206 SOA conditions), and conversely suppressive spike modulation (<0%) occurred relatively more frequently in the lower than in the upper groups. The difference in mean spike modulation between the two groups was statistically significant (11.49% for the upper vs -0.93% for the lower, two-sample t-test, p<10^−4^). Second, the frequency of RMS increase following S1-S2 sequence was greater in the upper (76.03%, 92 of 121 SOA conditions) than in the lower group (51.46%, 106 of 206 SOA conditions), and the mean LFP modulation differed significantly between the two groups (10.79 vs 0.27%, two-sample t-test, p<10^−6^). Thus, facilitation of spike and LFP response was dominant for the upper group, resulting in a stronger and more positive correlation between the two responses (r = 0.64), whereas both suppression and facilitation of spike and LFP response were equally observed for the lower group and the correlation between the magnitudes of spike and LFP responses was virtually absent (r = 0.03). Finally, the latency of the S2-evoked LFP change differed significantly, 41.57 ±5.23 ms for the upper, and 34.73 ±6.19 ms for the lower group (two-sample t-test, p<0.01). To summarize, in the upper group, surround stimuli tended to increase RMS power and facilitate the spike response, and these changes were coupled to each other. In contrast, in lower layers, the spike response tended to be relatively equally modulated by surround stimuli, and this change was variably coupled to the magnitude of LFP change.

**Fig 8:**
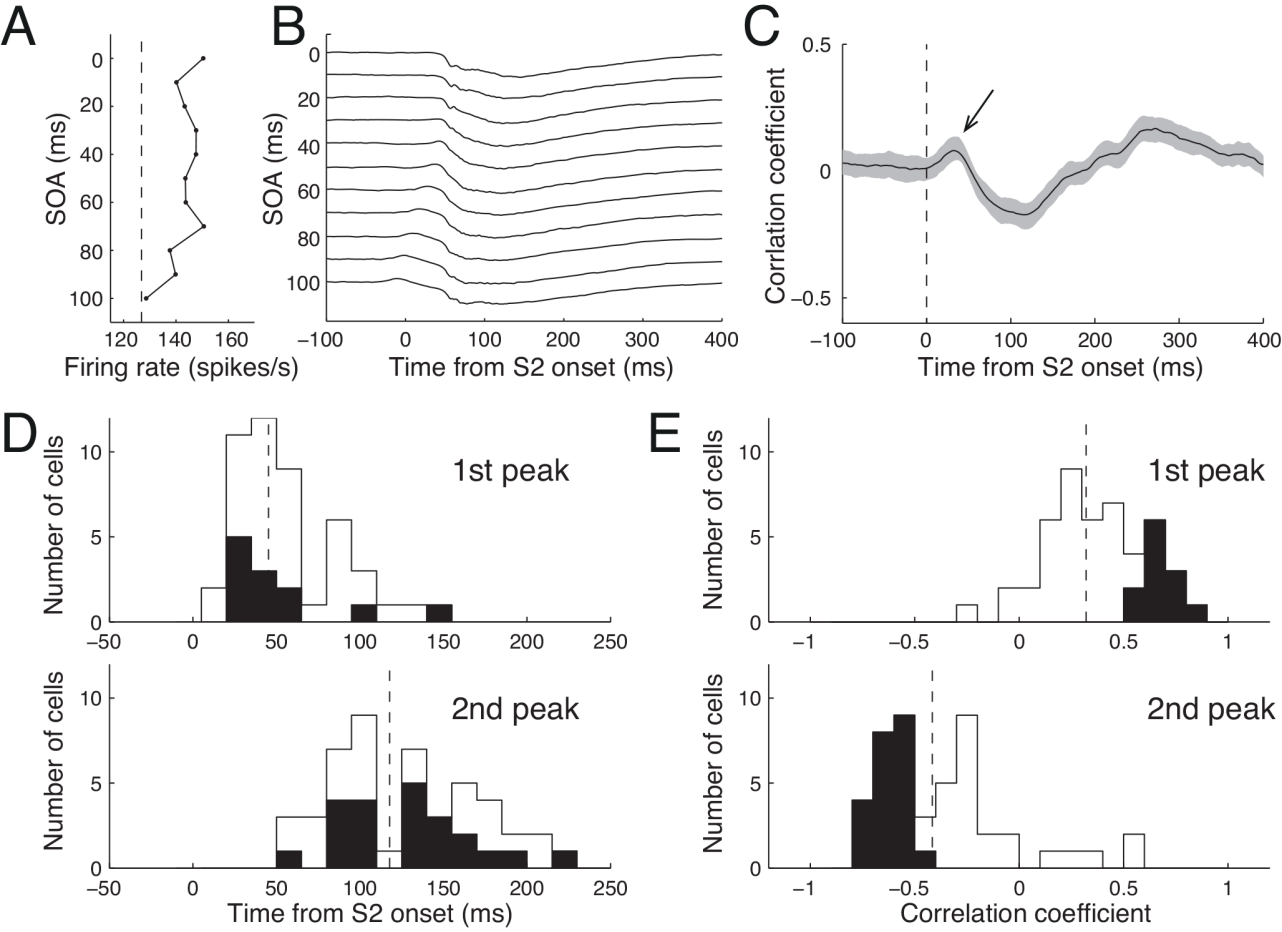
Correlation between spike activity and LFP. **A.** SOA-dependent spike modulation for the cell shown in Fig 3. The mean firing rates during the post-stimulus period of 50–150 ms of S2 are plotted as a function of SOA. Vertical dashed lines are the reference response levels evoked by S2 alone. **B.** Simultaneously recorded mean LFP traces in an arbitrary unit for corresponding SOAs for the cell shown in A. Traces are vertically shifted for visibility. **C.** Time course of mean correlation between spike and LFP modulation. The correlation coefficient between the SOA-dependent firing rate (as shown in A) and the instantaneous amplitude of LFP (as shown in B) was first calculated every 1 ms for each condition. Shown is the mean correlation coefficient time course averaged over all 517 stimulus conditions (11 SOAs X 47 S1-S2 sequences) from 31 cells including cases in which S1 was tested at more than one RF diameter away. The shading represents ±1 SE. Note a positive correlation immediately after S2 onset (arrow) and a subsequent negative correlation. **D, E.** Frequency histograms of the time from S2 onset (D) and the correlation coefficient (E) for the 1^st^ (upper) and 2^nd^ (lower) peaks in the time course of correlation. Dashed vertical lines indicate distribution means. For the 1^st^ peak correlation, the mean location was 45.25 ±36.0 ms and the mean correlation coefficient was 0.32 ±0.24. For the 2^nd^ peak, the mean location was 119.32 ±38.3 ms, and the mean correlation coefficient was -0.42 ±0.33. Black bars indicate significant cases, as determined with a bootstrap statistical test (p<0.05).

During S1-S2 sequence presentations, the S1-evoked sLFP is likely to mediate LFP modulation. However, its role in modulation of spike activity cannot be determined by the correlations shown in Fig 7. For that, we examined the time course of correlation between the magnitude of SOA-dependent LFP and that of spike activity (Fig 8). We first illustrate this analysis graphically with the SOA-dependency of spike activity (computed over a post-stimulus period of 50–150 ms after S2 onset, Fig 8A) and the SOA-dependent time course of LFP (Fig 8B) for the example cell shown in Fig 3. Note that the spike activity of the cell of Fig 3 was facilitated by S1-S2 sequence stimuli for most SOAs with respect to the response magnitude of the S2-alone condition (Fig 8A). The goal of this analysis was to temporally localize the pattern of SOA-dependency of spike activity on the trace of LFP signal. For the time course of correlation between the magnitude of SOA-dependent spike and corresponding LFP activity, the Pearson product-moment correlation coefficient was calculated based on 11 SOA conditions for each S1-S2 sequence condition, and averaged over all 47 sequence conditions tested at 31 recording sites. Overall, the correlation was initially positively peaking, but decreased until it reached a negative peak at around 100 ms after S2 onset (Fig 8C), a phenomenon that was mainly due to the upper group cells (not shown). The initial positive correlation occurred before the onset of spike activity in response to S2; the peak of correlation occurred at 33 ms after S2 onset (Fig 8C, arrow), and at 26 ms after S2 onset for upper groups cells. These results indicate that the raw LFP amplitude before onset of spike activity can predict the pattern of SOA-selectivity of spike activity that was calculated over the period of 50–150 ms after S2 onset.

We localized the first and second peaks of correlation (Fig 8D), and calculated the correlation coefficients at these peaks (Fig 8E) for each stimulus condition. For all 47 S1-S2 sequence conditions tested at 31 sites, the first and second peaks were found at 45.25 ±36.0 ms and 119.32 ±38.3 ms (dashed lines in Fig 8D). The mean correlation coefficients at these peaks were 0.32 ±0.24 and -0.42 ±0.33, respectively (Fig 8E). Again, the timing of the first peak correlation occurred before the peak spike activity, and even before the start of spike activity in some sequence conditions (Fig 8D, upper histogram). In other words, for these conditions, the pattern of SOA-dependency of LFP change predicted the SOA-dependency of spike modulation, even before the onset of spike activity, suggesting that the sLFP evoked by S1 influenced the spike modulation in response to S1-S2 sequence, consistent with Fig 8C. The timing of the second peak and its negative correlation suggest that this resulted from the peak spike activity being coupled to negative LFP [49] and a sharp negativity of the LFP at the time of the spike [50].

Since surround interaction involves suppression with varying temporal dynamics [51], we repeated the above analysis using different analysis windows for calculating SOA-dependency of spike activity: 50–200 ms and 50–300 ms after S2 onset. The results revealed similar locations and magnitudes of peak correlation, and the number of conditions with a significant correlation, 10 of 47 conditions for a positive first peak and 21 of 47 conditions for a negative second peak were significant with both analysis windows.

## Discussion

In the current study, we induced subthreshold LFPs with focal visual stimulus in the RF surround (S1). By its defining criterion employed in the current study, sLFP induced by S1 was virtually not contaminated by the spike activity at recording site, and thus enabled us to examine its effects on neural responses to ensuing optimal stimulus in the RF center (S2). Spike responses to the S1-S2 stimulus sequence differed from those evoked by S2 alone, in a manner that depended on the temporal interval (SOA) between the two stimuli. The SOA-dependent modulation of spike activity during the 50–150 ms interval after S2 onset was positively correlated with the instantaneous LFPs measured before spike initiation, an effect that was more prominent for cells in the upper cortical layers than for those in the lower layers. These results indicate that the sLFP evoked by S1 contributes to the modulation of spike activity, and that this contribution varies according to cortical depth.

### Propagation of sLFP

A focal visual stimulation triggers a wave of activity propagation on the surface of visual cortex [11, 13, 33, 38, 52]. In general, the electrical activity at one site in the brain can propagate to other sites in the form of action potentials along axonal arbors and across synapses. The current study was not designed to reveal the anatomical substrates of sLFP propagation. However, the pattern of non-linear decay of amplitude and linear increase in latency with distance in Fig 2 is consistent with the slow component of spike-triggered LFPs [17, 18, 33], and the propagation speed estimated from the results shown in Fig 2F and 2G suggests that the sLFP is a result of slow propagation through horizontal connections [11, 33, 52]. The dependence of the pattern of S1-induced LFP on cortical depth suggests involvement of synaptic mechanism.

We found that the sLFP was reliably evoked by stimuli falling as far as 10 mm away from the RF in cortical distance. This is much greater than estimates based on spike/stimulus-triggered LFPs [17–20, 33], but comparable to previous subthreshold intracellular responses evoked by peripheral stimuli as distant as 10–15° [7]. The sLFP showed decreased magnitude and increased latency as the cortical distance between S1 and S2 increased, consistent with previous studies on spike-triggered average of LFP signals [17, 18], suggesting that both these potentials arise through the same mechanism.

Bair and colleagues [6], employing large grating stimuli surrounding the RF at different distances from the RF, reported that the latency of spike suppression did not increase with distance, but depended on the strength of suppression, and suppression sometimes arrived faster than the excitatory RF response. However, a direct comparison with our results is difficult due to several differences in experimental protocol. First, latency was determined for the sLFP in our study, whereas they measured the latency of spike suppression. Second, in our study, S1s were identical in size and orientation, regardless of distance from the RF, whereas they manipulated center-surround distance by varying the inner diameter of full-field surround stimuli. Consequently, a nearer annulus occupied a larger visual space than a farther one in their study. We also note that for SOA of 0 and 10 ms, S1 and S2 temporally overlap, and we only speculate on the effect of this overlap. Since the off-response is faster than the on-response [53], interactions between the sLFP and spike activity may involve complex processes, especially for sequences with short SOAs. However, we noticed no apparent differences across SOA conditions in the correlation coefficients between geometric means of percentage change of LFP and spikes across trials, calculated separately for each SOA condition for either upper or lower group cells (not shown).

### Interaction between sLFP and response to RF stimulus

Previous studies have examined the cortical spread of the LFP and its relationship to spike activity [19, 20, 33, 43, 54, 55]. These studies, which were mostly based on the spike-triggered LFP, indicated that spike-LFP correlation decreases as stimulus size extends beyond the classical discharge field, suggesting a local nature of LFP in relation to multiple unit activity [19, 20]. In contrast, we specifically isolated a stimulus-locked LFP in V1. A major goal of the current study was to examine the roles of LFP in surround interaction by analyzing the interaction between the sLFP induced by a surround stimulus and the spike and LFP responses to an RF stimulus. In examining the relationship between spike and LFP signals, contamination between them has been a concern. In the current study, we isolated the LFP that was not contaminated by the spike activity at the recording site. To meet the criteria of ‘subthreshold’, one-third of the data were excluded during off-line analysis. This procedure enabled us to at least partly prevent LFP contaminated by the spiking itself from confounding the analysis. In addition, by temporally separating spike activity evoked by S2 and sLFP with the SOA, we further prevented spiking activity from contaminating LFP signal in determining the roles of LFP for spike response, as discussed below.

Introducing a variable SOA enabled us to examine the covariation between SOA-dependent modulation of LFP and spike activity. In S1-S2 sequence conditions, LFP is also influenced by spike activity, but after spike activity onset. Since S1 evokes subthreshold LFP, the LFP in the S1-S2 sequence condition is contributed by S1, especially during earlier phase before spike onset, and the contribution depends on SOA. Under the assumptions that the S1-induced sLFP collides with neural activity evoked by S2 with timing dictated by the SOA, and that this collision modulates spike activity, the SOA-dependency of spike activity (variable magnitude of spike response as a function of SOA) should mimic the SOA-dependency of LFP modulation. Indeed, the analyses of correlation between the magnitudes of LFP and spike modulation revealed a significant correlation for some neurons (Figs 7 and 8), substantiating this assumption, but this correlation appeared not based on the deviation from linear sum of LFPs evoked by S1 and S2 alone (Fig 6). The analysis of correlation also revealed the temporal relation between the two (Fig 8). In particular, the first positive correlation of Fig 8C and 8D suggests that modulation of LFP by S1 precedes spike modulation, suggesting a role of sLFP for spike modulation. The magnitude of the positive correlation was comparable to the ensuing negative correlation that was likely caused by spike-induced LFP changes (Fig 8E). Therefore, we conclude that the sLFP evoked by S1 participates in modulation of spike activity in response to S2.

It is known that the LFP and the membrane potential of cortical neurons in awake animals are correlated [56]. Also, particularly in awake animals, responses to visual stimulation are dominated by synaptic inhibition [57], and spike initiation is correlated with a decrease of inhibition [58]. We observed an asymmetry in the link between the LFP and spike activity; the facilitation of the spike response was associated with an increase in the RMS power of LFP, whereas the link between spike suppression and LFP change was not as evident as with spike facilitation (Fig 7). The LFP originates from extracellular currents of multiple sources including action potentials, excitatory currents, and inhibitory currents that may contribute differently to the induction of a LFP change. For example, inhibitory currents mediated by GABA_A_ are assumed to contribute little to field potentials [59].The asymmetry noted above may result from the interplay of such effects.

We observed that the pattern of interaction between the LFP and spike activity differs between the upper and lower depth groups. Since we did not use array type electrodes that simultaneously record from multiple sites spanning the full cortical depth, our depth estimates contain significant errors. We also note that the division of two depth groups was made with a criterion of 1.2mm below the surface of dura, which may be too shallow considering the thickness of dura and the space underneath. This probably reflects the fact that the tip of guide tube was positioned on the dura and then made gently pushing it down, but its precise position was likely to vary from day to day. However, regardless of the match between our depth division and anatomical laminar border, the roles of sLFP were depth-specific; surround stimuli evoked an sLFP in the upper cortical layers with large RMS power and facilitated the spike response in a manner suggesting systematic coupling to the LFP change, whereas at deeper cortical depths, sLFP power was relatively lower, and spike response tended to be suppressed. An additional negative peak that can be seen around 50 ms after S2 onset in Fig 7B has been noted to start at the border between supragranular and granular layers [48], and the relatively shorter LFP latency of the lower compared to upper group sites suggests that the border between the upper and lower depth groups is near layer 4C [29]. Thus, the upper group sites were mostly in the supragranular layers, and the group 2 sites mostly in the granular and infragranular layers. The depth-specific surround interaction has been studied in terms of feedforward versus feedback pathways [45], but direct comparison is difficult due to different stimulus configuration.

Previous studies have revealed various effects of surround stimuli, both suppressive and facilitative [3, 60–63], and conflicting results on the laminar effects of surround interactions, ranging from no significant laminar difference in surround suppression [64, 65] to laminar-specific surround suppression [66]. These inconsistent results may have been due to differences in the stimuli tested. Some studies used grating patches [65, 66], and others used grating annuli [45, 64] of varying contrast. We additionally introduced temporal aspects in the stimulus condition. Nevertheless, more facilitation in the upper and suppression in the lower depth group observed in the current study are consistent with previous reports [45, 66], and different surround interactions for the two depth groups are at least consistent with a recent report that cells of layers 2/3 show a greater contrast sensitivity to surround stimuli than to RF stimuli [51].

Spike modulation by surround stimulation is thought to enhance response selectivity [8–10] and integration of context information [42, 67, 68]. Signals evoked by S1 and S2 follow their own fixed time courses, and then disappear. Therefore, these signals can interact with each other only during a limited temporal window that is determined by the SOA between S1 and S2. However, as we pointed out in a previous study, this temporal window only partially explains the SOA-dependency of spike activity; spike modulation occurs within that temporal window, but even within the window, the magnitude of spike modulation varies with SOA [10]. Since surround interactions are mediated by multiple pathways, including feedforward, lateral, and feedback connections [2], one possibility is that S1 mediates LFP modulation via pathways that interact with each other even before modulating spike activity. The process determining LFP-dependency and SOA-dependency of spike modulation is intricate, and the precise relation between these components and sLFP in surround modulation awaits future studies.

## Supporting Information

S1 Fig Control of eye position. **A, B.** Eye position during the period of S2 presentation for the example cell of Fig 3. Each point is the mean eye position during the period of 20ms of S2 presentation in the conditions of S2-alone (A) and S1-S2 sequence with the SOA of 100ms (B). **C.** Horizontal (real) and vertical (dotted) eye positions during an example trial. Two vertical real lines indicate the times of S1 (earlier) and S2 (later) onset with the SOA of 30ms. Two vertical dashed lines indicate the analysis window of 300ms for counting microsaccades starting from -100ms to 200ms of S1 onset. **D.** Velocity of radial eye position with a threshold of 15 deg/s (horizontal dashed line). Four real and dashed vertical lines are same as in A. Three inverted triangles point the peak radial velocity of three movements that were taken as microsaccades. Two consecutive peaks with an inter-peak interval shorter than 100ms, such as shown near 800ms sometimes occurred, the first of which was considered as one microsaccade. **E.** Acceleration signal of radial eye position for determining onset and offset of microsaccades. **F.** Rate of microsaccade occurrence during the analysis window of 300ms across SOA conditions.
(EPS)
